# An Account of the Salutary Effects of a Salivation, and Also of Tonic Remedies, in Pulmonary Consumptions: In Three Letters to Dr. Edward Miller

**Published:** 1808-06

**Authors:** Benjamin Rush

**Affiliations:** Profesor of the Institutes of Medicine and of Clinical Practice in the University of Pennsylvania, &c. &c.


### *An* Account *of the* Salutary Effects *of a* Salivation, *and also of* Tonic Remedies, *in* Pulmonary Consumptions: *In Three Letters to* Dr. Edward Miller. *By* Benjamin Rush, *M. D. Professor of the Institutes of Medicine and of Clinical Practice in the University of Pennsylvania, &c. &c.*

### LETTER I.

Dear Sir,

On the 17th of December, 1800, William Kairns, a sailor, aged 26 years, was admitted into the Pennsylvania Hospital, in the second stage of Pulmonary Consumption. Several of this man's relations had died of that disease. As his pulse was active, I ordered eight ounces of blood to be drawn from his arm the day but one after his admission, and the same quantity to be taken five days afterwards. I directed him to live chiefly upon milk and vegetables, and to take a medicine, known in our hospital by the name of "Antimonial Powder," composed of fifteen grains of nitre, a sixth part of a grain of tartarized antimony, and half a grain of calomel, three times a day. Contrary to my intentions, sixteen doses of this powder brought on a salivation, the effects of which were as unexpected as they were agreeable. It suddenly put a stop to his cough, and removed every other symptom of his pulmonary disease. The salivation continued, without any additional doses of calomel, for ten days. I looked with a good deal of apprehension for a return of his consumptive symptoms after the spitting had ceased, but happily without finding them. In a week after his mouth and throat recovered from the mercurial disease; and he earnestly solicited his discharge, as a vessel was about to sail from our port, in which he was anxious to enter as a sailor. He was accordingly discharged, as cured, on the 10th of January, 1801.

On the 17th of January, 1801, William Poole, aged twenty-three years, formerly a merchant's clerk, was admitted into our hospital, in the third, or apparently the last stage of the disease which has been mentioned. His cough was distressing, especially in the nights. He was much emaciated, and had frequent chills, with constant sweats: his pulse forbad bleeding. Encouraged by the accidental cure just related, I prescribed for him the antimonial powder, and ordered mercurial ointment to be applied to his sides and breast, in order to excite a salivation. In a few days

I was

I was highly gratified, by hearing him complain of swelled gums, and great pains in his teeth. From this time, his cough, fever, chills and sweats left him. To obviate the weakness and want of appetite induced by his disease, I ordered him to take an infusion of columbo root, with elixir of vitriol, and to drink freely of wine and porter with his aliment, which I directed to be of a cordial nature, and from which he found great benefit. The salivary glands were not affected by the mercury in this patient; an effect of it which experience has taught me not to be essential in all cases to its salutary operation, in abstracting disease from internal and vital parts of the body. After this man recovered from his pulmonary symptoms, he was attacked by an obstinate and distressing rheumatism in one of his legs, which yielded to blisters, and the usual remedies for that form of disease. He was discharged, cured on the second of last May.

It was not in the winter 1801 that I first combated pulmonary consumption with mercury; I had frequently, many years ago, attempted to cure it by a salivation, but without being able to excite it. The late Dr. Thomas Bond, of this city, I have been informed, was equally unsuccessful with myself, in his attempts to cure this disease by the same revolutionary remedy. The knowledge that has been acquired by the more extensive and familiar use of mercury since our first pestilential year, 1793, has, I hope, taught me the cause of our failures. It was probably given before the morbid action of the blood-vessels was reduced by blood-letting and low diet; or, 2dly, after the suppurative action in the lungs was so far advanced, as to prevent the action of the mercury predominating over it. Mr. Bogue takes notice of the failure of mercury to excite a salivation in the diseases of the liver from the same cause. Perhaps a third cause of our want of success may be derived from our having giving the mercury, after all the excitability of the system (on which medicines act) had been wholly expended; or, 4thly, from our not giving it with some other medicine, calculated, by its previous or accompanying stimulus, to prepare the system for its impression upon the mouth or salivary glands; or, lastly, from our not giving it in a form, as in the antimonial powder, in which it is diffused through the mouth in the act of swallowing it.

I have said nothing of the state of the lungs in the above patients, previous to the healthy and happy change induced in them by the salivation. It is probable they were affected affected with tubercles or ulcers; both of which, I believe, have been cured by other remedies besides mercury; but if those consequences of pulmonary consumption did not exist, such a preternatural secretion and excretion of morbid matter had taken place in the lungs, as would have produced death as certainly as tubercles or ulcers when not opposed by medicine.

It is remarkable, that we have limited a salivation to an inflammation of the liver, as improperly as we have limited blood-letting to a pain in the side. There is no more reason why the former should not be prescribed as generally in diseases of all the internal and vital organs, than that the latter should not be used in diseases of great morbid action in every part of the body.

The success of this new mode of attacking pulmonary consumption will depend very much upon the time and manner of applying it. How long and how often have blood-letting, bark, opium, and blisters deceived us in the most simple cases, by our not regulating our prescriptions of them by the stage of the diseases in which they were indicated, or by the varying states of the system? It is from ignorance, or inattention to these two circumstances, that we often see such different issues of diseases from the use of the same remedies in the hands of different physicians.

I have only to add to my letter, that a patient should not be considered as safe or free from danger, after the removal of the cough, fever, and other symptoms of consumption by means of salivation. The general weakness which predisposed to it should be obviated by journies, sea voyages, a long course of tonics, and a total abstraction of all its remote and exciting causes.

*Philadelphia, March* 18, 1801.

---

### LETTER II.

DEAR SIR,

SOON after writing the above letter, an event occurred which made it necessary for me to delay sending it. William Kairns (whose case I have related,) in his passage down the Delaware, in the month of February, caught a severe cold, from being detained several weeks by the ice. He was sent back to our hospital on the second of March, where he died on the 20th of April following. Dr. Parke, who succeeded me in attending the hospital, politely attempted tempted to relieve him at my request by a salivation, but without success. If the death of this man should lead physicians to be more careful in enjoining their patients, after the cure of a pulmonary affection, to obviate the return of it, by avoiding all the remote and exciting causes of a relapse, and to use such remedies as are best calculated to remove the remaining debility of the system, it will not have occurred in vain. I have lately seen *Poole,* and was delighted to find him in good health and fine spirits. He had gained not only flesh, but fat.

Within the course of the present month, I have arrested this formidable disease, by gently touching the mouth with calomel, in two young ladies in our city, who were affected with alarming catarrhs, which had supervened previous debility of a long standing. They are both now riding in the country, by my advice, in order to remove the debility from their systems, which was the forerunner of their disease.

Dr. Stewart, an intelligent young physician of this city, has lately put into my hands, the history of a case of a Mrs. Cornvil, a woman of five and twenty years of age, whom he had cured in the spring of 1799, of a confirmed pulmonary consumption, by means of a salivation of five weeks continuance. The salivation, he remarks, was sometimes interrupted by a diarrhœa, and by great morbid excitement in the blood-vessels, but was always restored by checking the former by means of laudanum, and reducing the latter by means of blood-letting. The Doctor concludes his letter, which is dated May 12th of the present year, by saying, " Mrs. Cornvil is now free of all pulmonary complaints, and has, (since her cure,) become the mother of a fine healthy child." Dr. Physick informs me, he has salivated two patients in consumptions with success, and has hopes of curing a third, now under his care, by the same remedy. Thus have we made one more impression upon a most formidable and distressing disease. Let us not despair; and when we speak of an incurable disease, let us always remember, that we only express the imperfection of our knowledge in medicine.

*Philadelphia, May* 20, 1801.

---

LETTER

### LETTER III.

Dear Sir,

IN my last letters, I mentioned the effects of a salivation in pulmonary consumption. The subject of the present letter shall be, the effects of tonic remedies in that stage of consumption which forbids the use of depleting remedies, and in which mercury is ineffectual, from the exhausted state of the excitability of the system.

In the month of December, 1800, Benjamin Parker, aged 26 years, was admitted into the Pennsylvania Hospital, in the last stage of pulmonary consumption. He was unable to sit up in his bed. His expectoration was copious and purulent, and his pulse weak and frequent. Without any expectation of curing him, I prescribed a cordial stimulating diet, brandy toddy, liquid laudanum in small doses during the day, and from two to four grains of solid opium every night at bed time, and all chiefly with a view of smoothing his passage out of life. In my visits to the hospital after this prescription, I frequently passed his bed, without giving him the trouble of offering me his pulse. In the course of two weeks, Mr. Huchinson, one of the apothecaries of the hospital, informed me, with a smile, that Parker was much better. From this time I became more attentive to him, and had the pleasure of finding his countenance and pulse improving, and his cough lessening every day. By persevering in the use of the above remedies for six weeks, he recovered his strength and flesh, and was discharged on the 28th of February, without a single symptom of pulmonary consumption. This man had been a private patient of Dr. Caldwell before he was sent to our hospital. After his recovery, I asked the Doctor how long he thought he would live after he ceased to attend him? He replied, "not more than two weeks." I should have hesitated in communicating this case to you, had not every circumstance that has been related been witnessed by upwards of a hundred students of medicine who attended the practice of the hospital last winter. However extraordinary this cure may appear, it was not the first time I had seen similar good effects from tonic diet and medicines. I prescribed them for a sailor of the name of Prichard, in the spring of the year 1776, at his own house, and left him afterwards, expecting he would live but a few weeks. In a few months I saw him seated on the bench of one of the gondolas, which were employed to defend Philadelphia in the revolutionary war. We recollected each other. He informed me that he ate his allowance, did his duty as a gondolier, a gondolier, and slept every night in the open air upon a blanket under his bench. I saw him a year afterwards in the street in good health. Perhaps his exercise in rowing for several months on board the gondola, contributed to render his cure more complete.

If these two cases stood alone in the history of chronic diseases, they would be sufficient to teach us, never to abandon patients in cases apparently the most hopeless, without a parting effort to relieve them. They show farther, that the remedies which are calculated to lessen the pains which sometimes accompany the passage out of life, generally give the patient the best chance of recovery.

*Philadelphia, June* 19, 1801.

